# Novel Approach to Classify Plants Based on Metabolite-Content Similarity

**DOI:** 10.1155/2017/5296729

**Published:** 2017-01-09

**Authors:** Kang Liu, Azian Azamimi Abdullah, Ming Huang, Takaaki Nishioka, Md. Altaf-Ul-Amin, Shigehiko Kanaya

**Affiliations:** Graduate School of Information Science, Nara Institute of Science and Technology, 8916-5 Takayama, Ikoma, Nara 630-0192, Japan

## Abstract

Secondary metabolites are bioactive substances with diverse chemical structures. Depending on the ecological environment within which they are living, higher plants use different combinations of secondary metabolites for adaptation (e.g., defense against attacks by herbivores or pathogenic microbes). This suggests that the similarity in metabolite content is applicable to assess phylogenic similarity of higher plants. However, such a chemical taxonomic approach has limitations of incomplete metabolomics data. We propose an approach for successfully classifying 216 plants based on their known incomplete metabolite content. Structurally similar metabolites have been clustered using the network clustering algorithm DPClus. Plants have been represented as binary vectors, implying relations with structurally similar metabolite groups, and classified using Ward's method of hierarchical clustering. Despite incomplete data, the resulting plant clusters are consistent with the known evolutional relations of plants. This finding reveals the significance of metabolite content as a taxonomic marker. We also discuss the predictive power of metabolite content in exploring nutritional and medicinal properties in plants. As a byproduct of our analysis, we could predict some currently unknown species-metabolite relations.

## 1. Introduction

Plant taxonomy is the science that explores, describes, names, and classifies plants. The systematic and phylogenetic analysis of plants is traditionally based on macroscopic and microscopic morphological characteristics and is known to be turbulent [1]. The study of DNA and to a certain extent m-RNA and proteins has led to the immense subject of molecular biology, which has been increasingly applied to reconstruct the phylogeny of higher and lower plants [2]. The use of molecular data in plant taxonomy has been highly successful in many instances but has the following two limitations. First, current technologies that use genomic compartments instead of the entire genome data usually only partially reveal the evolutional relations among plants. The number of organisms with completely known genomes in Kyoto Encyclopedia of Genes and Genomes (KEGG) has now reached 4505 but includes only 65 plants (November 2016). This indicates that it is still impractical to reconstruct plant taxonomy using the entire genome information. Second, recent research has indicated that horizontal gene transfer occurs in multicellular eukaryotes, especially in plants, and has an important role in their eukaryotic evolution. This suggests that phylogenetic reconstruction cannot be determined conclusively from sequence data [3, 4]. Paralleled with molecular biology, exploration of the phylogenetic distance between species based on metabolites, either alone or in combination with sequence features, has also begun. Clemente et al. (2007) presented a method for assessing the structural similarity of metabolic pathways for several organisms and reconstructed phylogenies that were very similar to the National Center for Biotechnology Information (NCBI) taxonomy [5]. Borenstein et al. (2008) predicted the phylogenetic tree by comparing seed metabolite compound content [6]. Mano et al. (2010) considered the topology of pathways as chains and used a pathway-alignment method to classify species [7]. Chang et al. (2011) proposed an approach from the perspective of enzyme substrates and corresponding products in which each organism is represented as a vector of substrate-product pairs. The vectors were then compared to reconstruct a phylogenetic tree [8]. Ma et al. (2013) demonstrated the usefulness of the global alignment of multiple metabolic networks to infer the phylogenetic relationships between species [9]. However, most of these studies have focused on microorganisms, such as archaea, rather than multicellular eukaryotes.

Plants are the major contributors of natural products and are usually rich in nutritional or medicinal properties. Many natural products are biologically active and have been used for thousands of years as traditional medicines. Classifying plants on the basis of their chemical constituents, which is also known as plant chemosystematics, could be helpful in discovering new edible and medicinal plants and solving selected taxonomical problems [2, 10, 11]. Traditional chemosystematics of plants is based on the presence or absence of selected secondary metabolites, which is far from the holistic approach involving metabolite content [10, 11]. The incomplete data of metabolite constituents of plants limits the ability to solve taxonomical problems and discovery of new natural products or medicinal properties of plants.

With the rapid development of metabolomics, metabolite-related databases (DBs) have been created, including KNApSAcK, which contains accumulated information about species-metabolite relations including information about many secondary metabolites of plants [12]. Such information can be used in the systems-biological studies on the interactions between plants, including the activities of medicinal plants as well as interactions between plants and their environments [13]. Metabolite content refers to all small molecules that are the products or intermediates of metabolism (metabolites) that are present within a biological organism. The metabolite content of plants is dominated by secondary metabolites [14], which are usually of high structural diversity [15]. As a rule, secondary metabolites are often similar within members of a clade, and plants within a taxon often represent similar metabolite content and bioactive properties. Therefore, the metabolite content of plants can be used as a taxonomy marker to distinguish plants and other organisms [11]. However, the expression of secondary metabolites of a given structural type has frequently arisen on a number of occasions in different parts of the plant kingdom. This discrepancy could be due either to convergent evolution or to differential gene expression [11]. This suggests that the metabolite content of plants may reveal more information of the interaction and bioactive pattern of plants rather than morphology characteristics. Such metabolite-content-based classification not only reveals the phylogenetic relationship of plants but also can be used for studying the relationship of plants in terms of their bioactive properties, guiding prediction of medicinal properties in bioprospecting, exploring new nutritional or economic uses of plants, and solving taxonomical problems. Previously, microorganism species have been classified based on the volatile metabolites emitted by them, and the results have been well explained in terms of their pathogenicity [16]. This finding indicates that it is possible to classify other species, such as plants, based on metabolite-content similarity. With the development of plants metabolomics and big data biology, it is now possible to investigate the metabolite content of plants on a cross-class level [17, 18].

The KNApSAcK Core DB is an extensive plant-metabolite relation DB that can be applied in multifaceted plant research, such as identification of metabolites, construction of integrated DBs, and bioinformatics and systems biology [19, 20] and can be considered an advanced source of metabolite content of plants. The KNApSAcK Core DB contains 109,976 species-metabolite relationships that encompass 22,399 species and 50,897 metabolites, and these numbers are still growing [13]. In this paper, we propose an approach to classify plants based on metabolite-content similarity. The metabolite-content data of plants and structure data of compounds are mainly obtained from the KNApSAcK Core DB and partially from PubChem DB [21, 22]. We measure the structural similarity between two metabolites by using the concept of the Tanimoto coefficient [23, 24], construct a network by selecting highly structurally similar metabolite pairs, and determine structurally similar groups of metabolites by using the DPClus algorithm [25]. We then link plants to such metabolite groups instead of individual metabolites to represent the plants as binary vectors. Several structurally similar metabolites are generally involved in a metabolic pathway. Thus, the use of structurally similar metabolite groups in this study can help to reduce the effect of missing data. Next, the metabolite-content similarity between plants is calculated based on binary similarity coefficients which then transformed into metabolite-content distances. Plants are finally classified using the hierarchical clustering method, and the resulting classification is evaluated by comparing it with the NCBI taxonomy [26]. Our classification results reveal both the phylogeny- and bioactivity-based relations among plants. We also use a support vector machine (SVM) algorithm to classify the plants by their economic uses [27, 28]. The classification performance reveals the predictive power of metabolite content in exploring nutritional and medicinal properties of plants. As a byproduct of our analysis, we can predict some currently unknown species-metabolite relations. To the best of our knowledge, we are the first to classify plants based on metabolite content.

## 2. Materials and Methods

### 2.1. Dataset and Preliminaries

The major input data are species-metabolite relationships obtained from the KNApSAcK Core DB, which is a part of the KNApSAcK Family DB [13]. The KNApSAcK Core DB contains most of the published information about species-metabolite relations, but this is obviously far from complete regarding plants and other living organisms. In the preprocessing step, we removed the plants with inadequate plant-metabolite relations to guarantee that the amount of metabolite content of selected plants is sufficient enough to reveal their interrelations.

**Figure 1:**
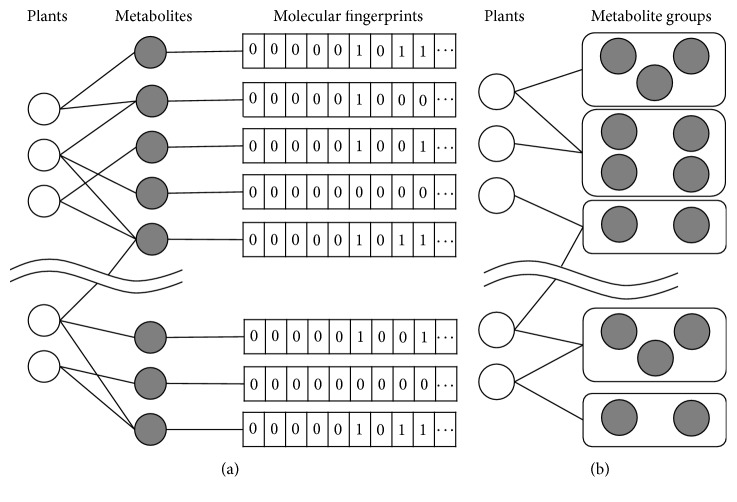
(a) Bipartite graph of plant-metabolite relations. Molecular structures of metabolites are described by 166-bit atom pair fingerprints, which are used to calculate Tanimoto structure similarity score for each metabolite pair. (b) Bipartite graph of plant versus metabolite-group relations. Each plant has been associated with metabolite groups instead of single metabolites to reduce effect of incomplete data.

We collected the molecular structure description files for the metabolites in our dataset as additional input data. The KNApSAcK Core DB provides MOL molecular structure files for most of the metabolites. For metabolite compounds with structure files that cannot be obtained from the KNApSAcK Core DB, we downloaded the SDF files directly from the PubChem DB [21, 22]. We used R package ChemmineR (v2.26.0) to generate atom pair fingerprints from molecular structure description files for all the metabolite compounds [29]. These molecular fingerprints were used to measure the structural similarity for all the metabolite pairs.  Figure 1(a) illustrates the binary plant-metabolite relations and corresponding molecular fingerprints.

### 2.2. Network Construction of Metabolites Based on Chemical Structure Similarity

Very little is known of the complete set of metabolite content of plants. Therefore, for classifying plants based on currently available metabolite-content data, an approach that can compensate for the limitations of missing data is needed. Adjacent metabolites along a metabolic pathway are often related to similar substructures; therefore, it can be assumed that structurally similar metabolites are involved in the same or similar pathway. Therefore, plants that share highly structurally similar metabolites are likely to have common pathways; thus, they are likely to be within the same category and represent similar bioactivity. To compensate for the gap in missing data, we primarily linked plants to structurally similar metabolite groups instead of individual metabolites for this study.

For the purpose of determining structurally similar metabolite groups, we initially constructed a network of metabolites based on chemical structure similarity. We used the Tanimoto coefficient to measure the structural similarity between two metabolites [23]. Willett (2014) investigated different structural similarity measures and concluded that chemoinformatics research on structural similarity would continue to be largely based on the use of 2D fingerprints, and the Tanimoto coefficient has been established as the standard for similarity searching [30]. The Tanimoto coefficient between two metabolites *A* and *B* is defined as follows, which is the proportion of the features shared by two compounds divided by their union:

(1) $$\begin{array}{c} Tanimoto\left(\;A,B\;\right)=\frac{AB}{A+B-AB}. \end{array}$$

The variable *AB* is the number of features common in both compounds, while *A* and *B* are the number of features that are related to the respective individual compounds. The Tanimoto coefficient has a range from 0 to 1 with higher values indicating greater similarity than lower ones. The Tanimoto coefficient can be calculated from molecular fingerprints using the R package ChemmineR [29]. Empirically, a Tanimoto coefficient value larger than 0.85 indicates that the compared compounds represent highly similar bioactive features [31]. We used 0.85 as the threshold to insert an edge between two metabolites and constructed a network of metabolites.

### 2.3. Clustering of Metabolites Based on DPClus

The DPClus algorithm is a graph-clustering algorithm that can be used to extract densely connected nodes as a cluster [25, 32]. This algorithm can be applied to an undirected simple graph *G* = (*N*, *E*) that consists of a finite set of nodes *N* and a finite set of edges *E*. Two important parameters are used in this algorithm (i.e., density *d* and cluster property cp). Density *d*_*k*_ of any cluster *k* is the ratio of the number of edges present in the cluster (|*E*|) to the maximum possible number of edges in the cluster (|*E*|_max_). The cluster property of a node *n* with respect to cluster *k* is represented as

(2) $$\begin{array}{c} {cp}_{nk}=\frac{E_{nk}}{d_{k}\times N_{k}}, \end{array}$$

where *N*_*k*_ is the number of nodes in *k* and *E*_*nk*_ is the total number of edges between *n* and each node of *k*.

In this study, we applied the DPClus algorithm to the structural similarity network of metabolites. The metabolites were divided into many groups such that each group contains structurally similar compounds and can be treated as a distinctive pattern of structure. Each metabolite group might be related to a certain pathway, which is related to the phylogeny and ecology of plants. A plant is related to a metabolite group if it is related to any metabolite in the group. Thus, the original plant-metabolite relations are transformed into plant versus metabolite-group relations, as shown in Figure 1(b). We used such groups to measure the similarity between plants, thus reducing the effects of incomplete metabolite-content data.

### 2.4. Clustering of Plants Based on Metabolite Groups

The relations between plants and structurally similar metabolite groups can be expressed with a sparse binary matrix, which is defined as *M*. Element *M*_*ij*_ = 1 means that plant *i* contains at least one metabolite of group *j*, and *M*_*ij*_ = 0 means that plant *i* contains no metabolite of group *j*. Therefore, for each plant, we obtain a binary vector such that each bit corresponds to the presence or absence of a metabolite group.

Let two plants be described by the binary vectors* x* and* y*, each comprised of *p* variables with values either 1 or 0 (“1” indicates presence while “0” indicates absence), and *p* is the total number of metabolite groups. The Simpson similarity coefficient between plants can be calculated as

(3) $$\begin{array}{c} S_{s}=\frac{a}{\min\left\{\left(a+b\right),\left(a+c\right)\right\}}. \end{array}$$

Here, *a*, *b*, and *c* are the frequencies of the events *x*&*y*, $x\&\overset{-}{y}$, and $\overset{-}{x}\&y$, respectively [33–35].

To strengthen our finding with more support, we also used the Jaccard coefficient, which was previously considered as a similarity measure between different organisms in different contexts [33, 36]. The Jaccard similarity coefficient can be calculated as

(4) $$\begin{array}{c} S_{j}=\frac{a}{a+b+c}. \end{array}$$

We transformed a similarity coefficient, *s*, to a distance coefficient, *d*, by the transformation *d* = 1 − *s* and classified the plants by using Ward's hierarchical clustering method using R.

### 2.5. Classification of Plants by SVMs

Support vector machines are supervised machine learning models for classification and regression analysis [27, 28]. An SVM training algorithm builds a model by constructing decision boundaries in feature space. Examples are predicted to belong to a category based on the boundaries.

To study the relationship between metabolite groups and economic uses of plants and evaluate the predictive power of metabolite content in guiding the discovery of natural products or medicinal properties in plants, we used an SVM algorithm, which was implemented by the function svm in R package e1071 v1.6-7, to classify plants by using default parameters [37–39]. We used economic uses as labels and corresponding metabolite groups as features. The classification performance is evaluated by using a confusion matrix. In a confusion matrix, the sum of a column represents the instances in a predicted class, while the sum of a row represents the instances in an actual class. All programs in this research were run in R v3.3.1.

## 3. Results and Discussion

### 3.1. Data Preprocessing

The KNApSAcK Core DB contains a total of 111199 species-metabolite binary relations that encompass 25658 species and 50899 metabolites. This DB was developed by collecting information on numerous metabolites of various organisms from published literature and several DBs, including PubChem [21, 22]. The species-metabolite relations in the KNApSAcK Core DB can be represented as a bipartite graph, as shown in Figure 1(a). The degree distribution of species in a species-metabolite bipartite graph follows a power law trend (see Supplementary Figure 1 of the Supplementary Material available online on https://doi.org/10.1155/2017/5296729) [40]. The metabolite-content data of plants in the KNApSAcK Core DB is unbalanced, i.e., many plants are associated with only a few metabolites and a few plants are associated with many metabolites, while other plants are in a between situation. One of the reasons behind this is that different plants have metabolic pathways of varying complexity. Medicinal plants usually contain more metabolites compared to edible plants because the former have gone through less artificial selections and preserved more secondary metabolites during evolution. Another reason is that the metabolomics of some important plants have been studied more systematically. The recorded metabolite content of such plants is more comprehensive compared to wild plants. Therefore, in our current research, we selected 216 plants from a total of 25658 plants in the KNApSAcK Core DB, such that each of the 216 plants is reported to be associated with no less than 30 metabolites, with 135 being the maximum number and 31 being the minimum. There are a total of 6522 metabolites related to the 216 plants in our input dataset.

### 3.2. Plant Representation Based on Metabolite-Content Similarity

**Figure 2:**
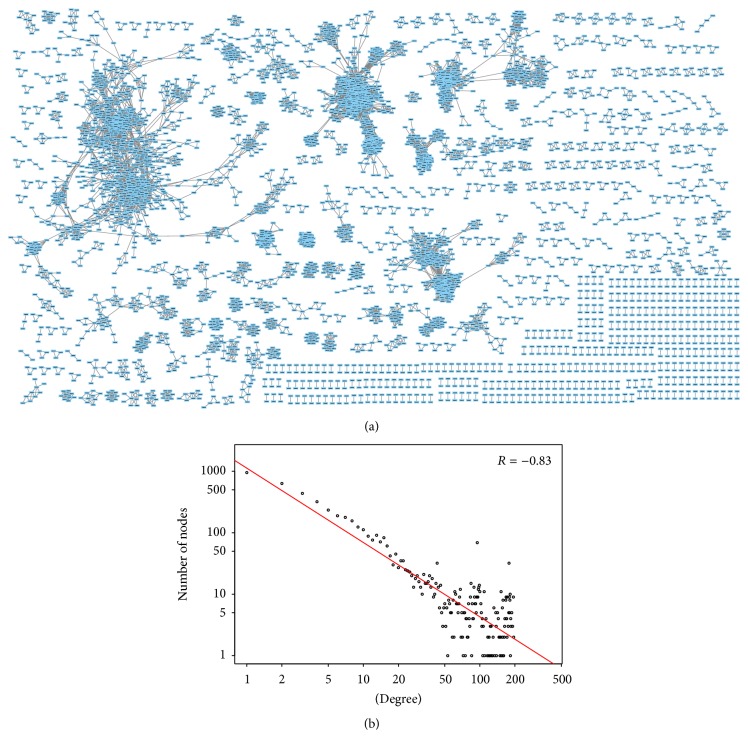
(a) Structural-similarity-based network of metabolites (plotted using network analysis tool* Cytoscape* v3.3.0). This network is composed of many isolated components, and each component contains different number of nodes. (b) Degree distribution of the network in log scale.

We dealt with 6522 metabolites involving 216 plants. We determined the Tanimoto coefficients between all possible metabolite pairs (21264981 pairs). We selected 54528 metabolite pairs with Tanimoto values greater than 0.85, which are 0.25% of all the metabolite pairs. On average, each metabolite is related to about eight different metabolites. We connected all the selected metabolite pairs and constructed a network of metabolites, as shown in Figure 2(a). This network involves 5085 metabolites and the other 1437 metabolites are not included in the network; that is, each of these metabolites is not structurally similar to any other metabolites. The 5085 metabolites included in the network are divided into 669 connected components. The degree distribution of the network also follows a power law trend (Figure 2(b)) [40].

To compensate for the gap in incomplete data regarding species-metabolite relations, we associated plants with structurally similar metabolite groups instead of individual metabolites. To achieve this, we applied the DPClus algorithm to the network of metabolites we developed, as discussed in the previous section. We did DPClus clustering with the following settings: cluster property cp was set to 0.5, density value *d* was set to 0.9, minimum cluster size was set to 2, and we used the overlapping mode.

**Figure 3:**
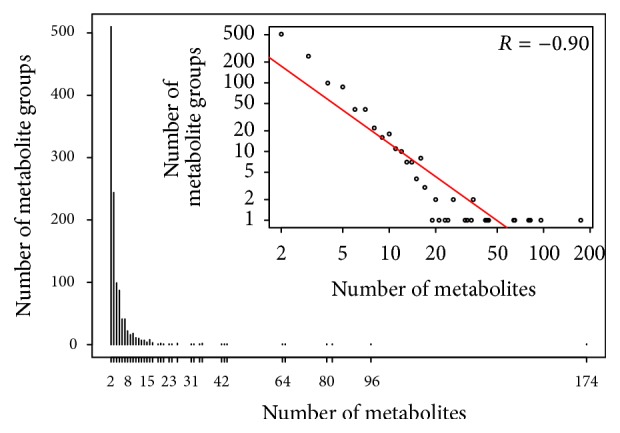
Frequency of metabolite groups with respect to group size.* x*-axes represent number of metabolites belonging to one metabolite group, and* y*-axes represent frequency of such metabolite groups. Frequency of metabolite groups in log scale is shown in inset figure.

The DPClus algorithm generated 1150 clusters (i.e., metabolite groups, involving 4700 metabolites). The largest group contained 174 metabolites, and there were 510 metabolite groups containing only 2 metabolites.  Figure 3 shows the frequency of metabolite groups with respect to size (the count of metabolites) in both normal scale and log-log scale (inset), and this distribution also follows a power law trend [40]. A total of 1822 metabolites not included in any cluster are considered as groups consisting of a single metabolite.

All clusters, large or small, contained structurally similar metabolites. Large clusters might be related to different metabolic pathways, but small clusters are likely related to specific metabolic pathways. A plant is related to a metabolite group if it is reported to contain any metabolite in the group. A plant can be represented as a binary vector such that each bit of the vector corresponds to the presence or absence of a metabolite group.

### 3.3. Clustering of Plants Based on Metabolite-Content Similarity

We calculated the plant-plant similarity by using two commonly used binary similarity coefficients Simpson and Jaccard [33]. The Jaccard coefficient has been used as a similarity measure to compare the enzyme content of metabolic networks in each pair of organisms [36]. The Simpson coefficient was devised to minimize the effect of the unequal size of two faunas being compared and having in the denominator only the number of taxa in a sample having the smaller number [34, 35].

We transformed a similarity score into a distance score *d* using *d* = 1 − *s* and then conducted Ward's hierarchical clustering analysis. Thus, we determined two dendrograms corresponding to two types of coefficients with our approach.

We used the NCBI taxonomy of the 216 plants generated using a web-based tool from the NCBI homepage (http://www.ncbi.nlm.nih.gov/taxonomy) as the reference classification [26]. The NCBI classification reflects the phylogenetic patterns within a plant group primarily based on morphology. According to the NCBI taxonomy, the 216 plants spread over 52 families with the largest family* Fabaceae* containing 42 plants.

We compared the dendrogram trees generated with our approach with the NCBI taxonomy based on a similarity score called Baker's Gamma correlation coefficient using R package* dendextend* v1.3.0 [41, 42]. Baker's Gamma correlation coefficient ranges from −1 to +1, with positive values, meaning that the two trees are statistically similar. The results show that both Simpson- and Jaccard-coefficient-based trees produced similar scores (i.e., 0.062 and 0.059, resp.), indicating that both trees are statistically similar with the NCBI taxonomy. We can also extract phylogeny relations from the trees by referring to the NCBI taxonomy.

Overall, we found that the Simpson coefficient performed better than the Jaccard coefficient. In the Simpson coefficient tree, more plants from the same genus or family appeared nearer to each other compared to the Jaccard coefficient tree. We illustrate this fact by pointing out some examples in Supplementary Figure 2. The better performance of the Simpson coefficient is also reflected with the Baker's Gamma correlation coefficient. Therefore, for further explanation, we selected the Simpson coefficient tree and classified the plants into 48 groups by cutting the dendrogram at variable threshold heights empirically chosen to enrich the clusters with plants of the same genus or family. Supplementary Figure 3 shows the dendrogram together with group IDs produced by our classification method.

The main defined ranks in the NCBI taxonomic hierarchy are as follows:* superkingdom*,* kingdom*,* phylum*,* subclass*,* order*,* family*,* subfamily*,* tribe*,* genus*, and* species *(from high to low). We collected the taxonomy information of 216 plants that we considered in this study and annotated each plant with ranks of* family* and* genus *(we used the scientific names of plants where the first word of a plant name represents the* genus* to which the plant belongs).  Table 1 lists the 48 groups of plants based on our clustering result with their taxonomic and usage information. The plants are arranged by different groups, and for each group plants within the same* family* or* genus* are arranged together to highlight the internal phylogeny relations. In the dendrogram of Supplementary Figure 3, neighboring plants belonging to the same* genus* or* family* are indicated by horizontal bold colored lines. Each* genus *or* family* is indicated by a specific color. It is evident that many clusters are rich with plants from the same* genus* or* family*. Thus, our results imply that plants in the same taxon correspond to similar metabolite content. Taking into account the inadequate amount of metabolite data and limited number of plants we considered for certain families, the results from our approach are very promising. These indicate that the proposed approach was designed to compensate for the shortcomings of limited data. Some deviations in our classification from the NCBI taxonomy can be explained in terms of ecological relationships or bioactive similarity. This implies that, compared to morphology-based taxonomy, metabolite-content-based classification reveals more information about the bioactive similarity among plants, which is related to the nutritional and medicinal properties of plants. Therefore, metabolite-content-based classification can be used as a time-efficient predictive tool for guiding discovery of edible and medicinal properties in wild plants.

### 3.4. Predicting Currently Unknown Plant-Metabolite Relations

The species-metabolite relation data in the KNApSAcK Core DB were collected from previously published papers. Many more plant-metabolite relations will inevitably be discovered in the future. However, based on our study, we can predict some not yet known plant-metabolite relations. When several plants are included in the same cluster with our approach, it implies that those plants contain many metabolites that are either the same or different but structurally very similar. When several plants contain a different subset of a group of structurally similar metabolites and they are very close according to morphological taxonomy, we can assume that all those plants contain the union of the metabolites currently detected in them. The basis of this assumption is that similar metabolic pathways are expected to be active in plants within a given taxon group.

In our experiments, we found structurally similar metabolite groups of different sizes, large and small. However, the metabolites belonging to a smaller group are likely to be closely related along a certain metabolic pathway. Therefore, for predicting currently unknown plant-metabolite relations, we focused on only smaller metabolite groups and empirically considered the metabolite groups of size no more than eight.

In summary, we follow the following steps to improve prediction accuracy.

**Step 1 .**

We select a group of plants that are in the same cluster according to our approach and at the same time belong to the same genus or family. Let us call such a group *S*.

**Step 2 .**

We determine the set (*K*) of structurally similar metabolite groups of size no more than eight such that each metabolite group is associated with at least two plants in *S*.

**Step 3 .**

All the metabolites of a metabolite group in *K* are assigned to the plants in* S* which are associated with the group. This process is repeated for each group in *K*.

Based on known information, however, we exclude some metabolites that are mainly structure isomers from this prediction process because some isomers are usually produced by different pathways [43, 44]. We discuss this method with an example as follows.

*Predicting Metabolites for Citrus Plants*. Six* Citrus* plants* (Citrus limon, Citrus aurantifolia*,* Citrus paradisi*,* Citrus sinensis*,* Citrus reticulata*, and* Citrus aurantium)* are considered an excellent group in our classification (Group 1 in Table 1, we call it group *S*) and belong to the same genus* (Citrus)*. We extract the set *K* of metabolite groups (with size no more than eight) in which each metabolite group is associated with at least two plants in *S*. There is a total of 58 such metabolite groups in *K*. For each metabolite group in *K* which is related to multiple plants, we can construct a plant-metabolite table.  Table 2 is a plant-metabolite table for a given metabolite group that contains two metabolites,* Limonene* and* Cyclohexane*, and their association to six plants in *S*. In Table 2, “1” means that the metabolite is reported in the corresponding plant and “0” means that the metabolite is unreported in that plant. We treat all these unreported plant-metabolite relations as currently unknown but actual relations. We repeat this process for all 58 metabolite groups in *K* and obtain a list of unrecorded metabolites for the plants in *S*, which we show in Table 3. Following this method, we can predict some currently unrecorded metabolites and find some widespread medicinal species that can be substitutions of more endangered relatives currently being used [45].

Not all the predicted metabolites might actually be produced in given plants because of the complexity of metabolic pathway evolution. On the contrary, many true relations could not be predicted due to the limitation of the incomplete data source. However, with developments in plant metabolomics, we may be able to add more plant-metabolite relations in our analysis in the future and produce better results. For other plant groups, we can also predict numerous unrecorded metabolites. We list all the predicted plant-metabolite relations in Supplementary Table 1.

### 3.5. Relationship between Metabolite Content and Uses of Plants

Our unsupervised approach for classifying plants is based on metabolite-content similarity using hierarchical clustering. Our results substantially match those of traditional morphology-based taxonomy. However, our results further reflect the usage patterns of plants.

The metabolite content of plants is always related to their bioactive properties, and the similarity of the metabolite content of plants can reveal their bioactive similarity. Generally, medicinal properties are not randomly distributed in different classes of plants. Some plant classes are represented by more medicinal plants than others. It is suggested that there is a phylogenetic pattern in medicinal properties even within one genus [45–47]. A similar distribution could also be observed in our classification that plants with certain uses are concentrated in the same group. Many plant groups in our classification are of similar usage patterns. A plant is frequently related to multiple uses, but we only consider the most common use in this paper. We collected all the plant resource information from various data sources, including Wikipedia (https://www.wikipedia.org), and annotated plants by their uses such as medicinal, edible, ornamental, forestry, poisonous, and timber.  Table 1 lists the usage patterns of 216 plants. The economic uses of plants are represented by different letters (E: edible, M: medicinal, L: landscaping, including forestry and ornamental plants, T: timber, P: poisonous, and W: wild plants that are not yet widely used by humans). Eleven groups (ID: 1, 9, 10, 12, 13, 14, 15, 19, 26, 39, and 40) involving 38 plants mostly consist of edible plants, and 14 groups (ID: 2, 4, 6, 18, 21, 27, 29, 31, 36, 38, 41, 43, 44, and 48) involving 69 plants mostly consist of medicinal plants. Moreover, 3 groups (ID: 8, 17, and 30) involving 10 plants mostly consist of landscaping or timber plants. This implies that the proposed classification approach of plants is consistent with their economic uses.

In this section, we investigate the relations between usage patterns and metabolite content of plants using a supervised classification technique. We considered every metabolite group as a pathway pattern such that each group can be used as a feature for classifying plants by their uses. For this analysis, we considered 48 edible plants (E), 81 medicinal plants (M), 14 timber plants (T), 14 landscaping plants (including forestry and ornamental plants), and 5 poisonous plants (P). We considered the plants that have both edible and medicinal uses (plants with “E/M” in Table 1) as medicinal plants. We applied an SVM algorithm to classify the plants, using economic uses of plants as labels and corresponding metabolite groups as features. Classification performance was evaluated from the resulting confusion matrix, as shown in Table 4. The rows of the confusion matrix indicate documented uses of plants and columns indicate the predicted uses from the SVM algorithm.* Recognition rate *is the proportion of correctly predicted plants corresponding to a class.

We found that all the medicinal plants and all but one edible plant were classified correctly. This implies that the metabolite content of medicinal and edible plants substantially differs. However, half the timber and landscaping plants were classified as medicinal plants. Therefore, timber and landscaping plants are somewhat related to medicinal plants in terms of metabolite content. All the poisonous plants were classified incorrectly: four plants were classified as medicinal plants and one as edible. This implies that poisonous plants are more similar to medicinal plants. Many poisonous plants can be used in treating specific diseases if the doses are carefully controlled [48]. In summary, edible plants represent exclusive metabolite content and can be differently classified from inedible plants. Furthermore, metabolite-content-based classification also reveals the predictive power of medicinal properties in bioprospecting. This indicates that our proposed approach can be used for exploring nutritional or medicinal properties of plants.

## 4. Conclusion

We proposed an approach for comparing the metabolite content of plants and classifying plants by their metabolite content. We showed that with this approach we can classify plants similar to the traditional morphology-based plant taxonomy. Naturally, this work can be generalized from various perspectives. First, our approach can be regarded as a novel chemosystematics method that can be used to consider the global metabolite content of plants instead of a group of metabolites as done in previous research. The resulting classification is consistent with natural phylogenetic and chemosystematics patterns of plants. Some deviations in our classification from the NCBI taxonomy can be explained in terms of bioactive similarity. Moreover, the complexity and known extent of metabolite content vary for different plants. We found that the Simpson coefficient can minimize the effect of the unequal size of the metabolite content of organisms and performs better in comparing metabolite content of plants than the Jaccard coefficient, which has been widely used as a similarity measure in various biological studies.

We also described a method for predicting unrecorded metabolites by structurally similar metabolite groups and phylogenetic relation of plants. With this method, we can predict some unrecorded metabolites and find new edible/medicinal plants from wild plants that have not been used by humans. Moreover, we studied the relation between the metabolite content of plants and their economic uses. We found that edible and medicinal plants represent unique metabolic pathway patterns and can be classified with an SVM algorithm with our integrated metabolite-content data. Our proposed metabolite-content-based plant-classification approach reveals the predictive power of medicinal properties in bioprospecting. The performance of this approach depends on the completeness of the metabolite-content data we use because metabolite groups, which were regarded as metabolic pathway patterns in our research, have been extracted from the background network of metabolites by using the DPClus algorithm. Therefore, if we can add more plant-metabolite relations, we can classify metabolites and species more accurately. Also, metabolites along identical pathways always correspond to high structural similarity. Our approach will be useful for predicting metabolic pathways in plants.

## Supplementary Material

Supplementary Material includes 3 Supplementary Figures and 1 Supplementary Table. The Supplementary Figures contain the distribution of species in species-metabolite bipartite graph (Supplementary Figure 1), the comparision of Simpson- and Jaccard-coefficient-based dendrograms (Supplementary Figure 2), and phylogeny patterns in proposed classification of plants (Supplementary Figure 3). The Supplementary Table contains a list of predicted plant-metabolite relations (Supplementary Table 1).

## Figures and Tables

**Table 1:** Taxonomic and use information of 216 plants. Group ID, plant names, taxonomic ranks (*family*), and economic uses are mentioned in consecutive columns. Economic uses of plants are represented as the following abbreviations: E (edible), M (medicinal), L (landscaping,), T (timber), P (poisonous), and W (wild plant). Some plants are both edible and medicinal and are annotated as E/M.

Group	Plant	Family	Use
1	*Citrus limon*	Rutaceae	E
	*Citrus aurantifolia*	Rutaceae	E/M
	*Citrus paradisi*	Rutaceae	E
	*Citrus sinensis*	Rutaceae	E
	*Citrus reticulata*	Rutaceae	E
	*Citrus aurantium*	Rutaceae	E

2	*Houttuynia cordata*	Saururaceae	E/M
	*Houttuynia emeiensis*	Saururaceae	W
	*Rhodiola rosea*	Crassulaceae	M

3	*Artemisia annua*	Asteraceae	M
	*Artemisia capillaris*	Asteraceae	M
	*Rhaponticum carthamoides*	Asteraceae	W
	*Solanum lycopersicum*	Solanaceae	E

4	*Anthemis aciphylla*	Asteraceae	W
	*Artemisia annua *L.	Asteraceae	M
	*Centaurea sessilis*	Asteraceae	W
	*Valeriana officinalis*	Caprifoliaceae	M
	*Persicaria minus*	Polygonaceae	M
	*Mentha arvensis*	Lamiaceae	M
	*Peucedanum paniculatum*	Apiaceae	W

5	*Zingiber officinale*	Zingiberaceae	E/M
	*Alpinia galanga*	Zingiberaceae	E/M
	*Rosmarinus officinalis*	Lamiaceae	M
	*Cistus albidus*	Cistaceae	W
	*Pinus halepensis*	Pinaceae	L

6	*Myrtus communis*	Myrtaceae	M
	*Leptospermum scoparium*	Myrtaceae	M
	*Santolina corsica*	Asteraceae	W

7	*Curcuma amada*	Zingiberaceae	E/M
	*Curcuma aeruginosa*	Zingiberaceae	W
	*Cistus creticus*	Cistaceae	W
	*Melaleuca leucadendra*	Myrtaceae	M
	*Piper arboreum*	Piperaceae	W
	*Piper fimbriulatum*	Piperaceae	W
	*Cedrus libani*	Pinaceae	L
	*Cyperus rotundus*	Cyperaceae	M

8	*Pseudotsuga menziesii*	Pinaceae	T
	*Pinus sylvestris*	Pinaceae	T
	*Picea abies*	Pinaceae	T
	*Citrus unshiu*	Rutaceae	E

9	*Prunus persica*	Rosaceae	E
	*Prunus avium*	Rosaceae	E
	*Prunus cerasus*	Rosaceae	E

10	*Pisum sativum*	Fabaceae	E
	*Lathyrus odoratus*	Fabaceae	L
	*Allium cepa*	Amaryllidaceae	E

11	*Linum usitatissimum*	Linaceae	T
	*Vicia faba*	Fabaceae	E
	*Carthamus tinctorius*	Asteraceae	M

12	*Phaseolus lunatus*	Fabaceae	E
	*Phaseolus vulgaris*	Fabaceae	E
	*Phaseolus coccineus*	Fabaceae	E

13	*Triticum aestivum*	Poaceae	E
	*Zea mays*	Poaceae	E
	*Spinacia oleracea*	*Amaranthaceae*	E

14	*Raphanus sativus*	Brassicaceae	E
	*Brassica napus*	Brassicaceae	P
	*Malus domestica*	Rosaceae	E

15	*Hordeum vulgare*	Poaceae	E
	*Oryza sativa*	Poaceae	E
	*Cucumis sativus*	Cucurbitaceae	E
	*Glycine max*	Fabaceae	E
	*Helianthus annuus*	Asteraceae	E

16	*Eriobotrya japonica*	Rosaceae	E
	*Cassia fistula*	Fabaceae	M
	*Aesculus hippocastanum*	Hippocastanaceae	P
	*Camellia sinensis*	Theaceae	E
	*Rheum *sp.	Polygonaceae	W

17	*Robinia pseudoacacia*	Fabaceae	L
	*Colophospermum mopane*	Fabaceae	T
	*Acacia mearnsii*	Fabaceae	W

18	*Sinocrassula indica*	Crassulaceae	M
	*Sedum sarmentosum*	Crassulaceae	M
	*Rhodiola sachalinensis*	Crassulaceae	M
	*Phyllanthus emblica*	Phyllanthaceae	E/M
	*Psidium guajava*	Myrtaceae	E
	*Phellodendron amurense*	Rutaceae	M
	*Epimedium sagittatum*	Berberidaceae	M

19	*Solanum lycopersicum*	Solanaceae	E
	*Solanum tuberosum*	Solanaceae	E
	*Nicotiana tabacum*	Solanaceae	M

20	*Capsicum annuum*	Solanaceae	E
	*Petunia x hybrida*	Solanaceae	L
	*Daucus carota*	Apiaceae	W
	*Asclepias curassavica*	Apocynaceae	L
	*Humulus lupulus*	Cannabaceae	M
	*Cyperus rotundus*	Cyperaceae	M

21	*Glycyrrhiza uralensis*	Fabaceae	M
	*Glycyrrhiza aspera*	Fabaceae	W
	*Glycyrrhiza glabra*	Fabaceae	E/M
	*Glycyrrhiza inflata*	Fabaceae	M

22	*Lupinus luteus*	Fabaceae	W
	*Lupinus albus*	Fabaceae	E
	*Derris scandens*	Fabaceae	W
	*Erythrina variegata*	Fabaceae	L
	*Erythrina senegalensis*	Fabaceae	M

23	*Euchresta japonica*	Fabaceae	W
	*Euchresta formosana*	Fabaceae	W
	*Sophora flavescens*	Fabaceae	M
	*Maackia amurensis*	Fabaceae	L
	*Sophora secundiflora*	Fabaceae	W
	*Daphniphyllum oldhamii*	Daphniphyllaceae	M

24	*Medicago sativa*	Fabaceae	E
	*Clitoria ternatea*	Fabaceae	E
	*Trifolium pratense*	Fabaceae	M
	*Sophora japonica*	Fabaceae	T
	*Lespedeza homoloba*	Fabaceae	W
	*Melilotus messanensis*	Fabaceae	W
	*Glycyrrhiza pallidiflora*	Fabaceae	W
	*Dalbergia odorifera*	Fabaceae	T

25	*Corydalis claviculata*	Papaveraceae	W
	*Papaver somniferum*	Papaveraceae	M
	*Corydalis solida*	Papaveraceae	W
	*Cocculus laurifolius*	Menispermaceae	W
	*Stephania cepharantha*	Menispermaceae	W
	*Stephania cepharantha*	Menispermaceae	W
	*Cocculus pendulus*	Menispermaceae	W
	*Annona cherimola*	Annonaceae	E
	*Xylopia parviflora*	Annonaceae	W

26	*Brassica oleracea*	Brassicaceae	E
	*Brassica rapa*	Brassicaceae	E
	*Armoracia lapathifolia*	Brassicaceae	E
	*Hesperis matronalis*	Brassicaceae	L

27	*Alstonia macrophylla*	Apocynaceae	T
	*Alstonia angustifolia*	Apocynaceae	M
	*Alstonia angustifolia *var. *latifolia*	Apocynaceae	M

28	*Millettia pinnata*	Fabaceae	L
	*Millettia pinnata*	Fabaceae	L
	*Neorautanenia amboensis*	Fabaceae	W
	*Tephrosia purpurea*	Fabaceae	P
	*Amorpha fruticosa*	Fabaceae	L
	*Piscidia erythrina*	Fabaceae	T

29	*Gymnadenia conopsea*	Orchidaceae	M
	*Bletilla striata*	Orchidaceae	M

30	*Taiwania cryptomerioides*	Cupressaceae	T
	*Chamaecyparis formosensis*	Cupressaceae	T
	*Cryptomeria japonica*	Cupressaceae	T

31	*Gutierrezia microcephala*	Asteraceae	P
	*Saussurea lappa*	Asteraceae	M
	*Artemisia *spp.	Asteraceae	W
	*Citrus *spp.	Rutaceae	E
	*Citrus sudachi*	Rutaceae	M
	*Murraya paniculata*	Rutaceae	M
	*Cannabis sativa*	Cannabaceae	M
	*Iris domestica*	Iridaceae	M

32	*Tabernaemontana coffeoides*	Apocynaceae	W
	*Kopsia dasyrachis*	Apocynaceae	W
	*Catharanthus roseus*	Apocynaceae	M
	*Rauvolfia vomitoria*	Apocynaceae	W

33	*Nardostachys chinensis*	Caprifoliaceae	W
	*Acritopappus confertus*	Asteraceae	W
	*Isodon xerophilus*	Lamiaceae	W
	*Cynanchum sublanceolatum*	Apocynaceae	W
	*Caesalpinia crista*	Fabaceae	T
	*Murraya euchrestifolia*	Rutaceae	W
	*Curcuma zedoaria*	Zingiberaceae	E

34	*Garcinia mangostana*	Clusiaceae	E/M
	*Garcinia dulcis*	Clusiaceae	W

35	*Atalantia buxifolia*	Rutaceae	W
	*Ruta graveolens*	Rutaceae	E/M
	*Clausena excavata*	Rutaceae	W
	*Angelica furcijuga*	Apiaceae	E/M

36	*Andrographis paniculata*	Acanthaceae	M
	*Scutellaria baicalensis*	Lamiaceae	M

37	*Zanthoxylum simulans*	Rutaceae	M
	*Zanthoxylum integrifolium*	Rutaceae	W

38	*Magnolia denudata*	Magnoliaceae	M
	*Magnolia officinalis*	Magnoliaceae	M
	*Aeschynanthus bracteatus*	Gesneriaceae	W

39	*Broussonetia papyrifera*	Moraceae	E
	*Morus alba*	Moraceae	E/M
	*Artocarpus communis*	Moraceae	E

40	*Sinapis alba*	Brassicaceae	E
	*Vachellia rigidula*	Fabaceae	E

41	*Lycium chinense*	Solanaceae	M
	*Mandragora autumnalis*	Solanaceae	M
	*Angelica sinensis*	Apiaceae	M

42	*Cullen corylifolium*	Fabaceae	M
	*Calophyllum inophyllum*	Calophyllaceae	T
	*Juniperus phoenicea*	Cupressaceae	W

43	*Taxus cuspidata*	Taxaceae	P
	*Taxus brevifolia*	Taxaceae	M
	*Taxus baccata*	Taxaceae	M
	*Taxus wallichiana*	Taxaceae	M
	*Taxus chinensis*	Taxaceae	M
	*Taxus mairei*	Taxaceae	M
	*Taxus yunnanensis*	Taxaceae	M

44	*Panax notoginseng*	Araliaceae	M
	*Panax ginseng*	Araliaceae	M
	*Panax pseudoginseng *var*. notoginseng*	Araliaceae	M
	*Panax ginseng *C.A. Meyer	Araliaceae	M
	*Bupleurum rotundifolium*	Apiaceae	M
	*Beta vulgaris*	Amaranthaceae	E
	*Bellis perennis*	Asteraceae	E/M

45	*Xylocarpus granatum*	Meliaceae	W
	*Spiraea formosana*	Rosaceae	W
	*Hibiscus taiwanensis*	Malvaceae	W
	*Begonia nantoensis*	Begoniaceae	W
	*Alpinia blepharocalyx*	Zingiberaceae	W
	*Taraxacum formosanum*	Asteraceae	W

46	*Aristolochia elegans*	Aristolochiaceae	L
	*Aristolochia heterophylla*	Aristolochiaceae	M

47	*Artabotrys uncinatus*	Annonaceae	W
	*Annona purpurea*	Annonaceae	E
	*Rubia yunnanensis*	Rubiaceae	M
	*Withania somnifera*	Solanaceae	M

48	*Salvia officinalis*	Lamiaceae	E/M
	*Orthosiphon stamineus*	Lamiaceae	W
	*Plantago major*	Plantaginaceae	M
	*Rehmannia glutinosa*	Rehmanniaceae	M
	*Olea europaea*	Oleaceae	E/M
	*Lonicera japonica*	Caprifoliaceae	M
	*Eleutherococcus senticosus*	Araliaceae	M
	*Diospyros kaki*	Ebenaceae	E
	*Punica granatum*	Lythraceae	E
	*Curcuma domestica*	Zingiberaceae	E/M

**Table 2:** Reported plant-metabolite relations of 6 plants of genus *Citrus* with a given metabolite group (including 2 metabolites: *Limonene* and *Cyclohexane*). 1/0 indicates presence/absence of a metabolite in a plant.

	*Citrus limon*	*Citrus aurantifolia*	*Citrus paradisi*	*Citrus sinensis*	*Citrus reticulata*	*Citrus aurantium*
Limonene	1	1	1	1	1	1
Cyclohexane	0	1	1	1	1	0

**Table 3:** Predicted unrecorded metabolites for 6 *Citrus* plants, encompassing 38 plant-metabolite relations.

Species	Predicted unrecorded metabolites
*Citrus limon*	Gibberellin A4; methyl salicylate; cyclohexane; *o*-isopropenyl toluene; jasmonic acid; 10′-apoviolaxanthal; alpha-trans-bergamotene
*Citrus aurantifolia*	Methyl salicylate; citral; benzeneacetaldehyde; *o*-isopropenyl toluene; methyl epijasmonate; salvigenin
*Citrus paradisi*	Rhoifolin; isopropanol; methyl salicylate; citral; benzeneacetaldehyde; *o*-isopropenyl toluene
*Citrus sinensis*	Isoscutellarein 7,8-dimethyl ether; isoscutellarein 7,8,4′-trimethyl ether; *o*-isopropenyl toluene; methyl epijasmonate; salvigenin; gibberellin A53; violaxanthin
*Citrus reticulata*	Gibberellin A81; gibberellin A9; isopropanol; citral; 6-demethoxytangeritin; tetramethylscutellarein
*Citrus aurantium*	Apigenin 7-rutinoside; methyl salicylate; salvigenin; cyclohexane; benzeneacetaldehyde; *o*-isopropenyl toluene

**Table 4:** Resulting confusion matrix from support vector machine (SVM) algorithm. 162 plants are labeled as edible (E), medicinal (M), timber (T), landscaping (L), and poisonous (P), and SVM model was constructed to classify them.

	M	E	T	L	P	Recognition rate [%]
M	81	0	0	0	0	100
E	1	47	0	0	0	97.9
T	6	0	8	0	0	57.2
L	8	1	0	5	0	35.7
P	4	1	0	0	0	0

Total: 162 plants. Accuracy: 87.0%.
