# Supplementary material for: Novel Approach to Classify Plants Based on Metabolite-Content Similarity
Source: Biomed Res Int. 2017 Jan 9;2017:5296729. doi: 10.1155/2017/5296729 (PMC5253511; doi:10.1155/2017/5296729)
Supplement: Supplementary file 1 — Supplementary Material includes 3 Supplementary Figures and 1 Supplementary Table. The Supplementary Figures contain the distribution of species in species-metabolite bipartite graph (Supplementary Figure 1), the comparision of Simpson- and Jaccard-coefficient-based dendrograms (Supplementary Figure 2), and phylogeny patterns in proposed classification of plants (Supplementary Figure 3). The Supplementary Table contains a list of predicted plant-metabolite relations (Supplementary Table 1). [file 5296729.f1.pdf]

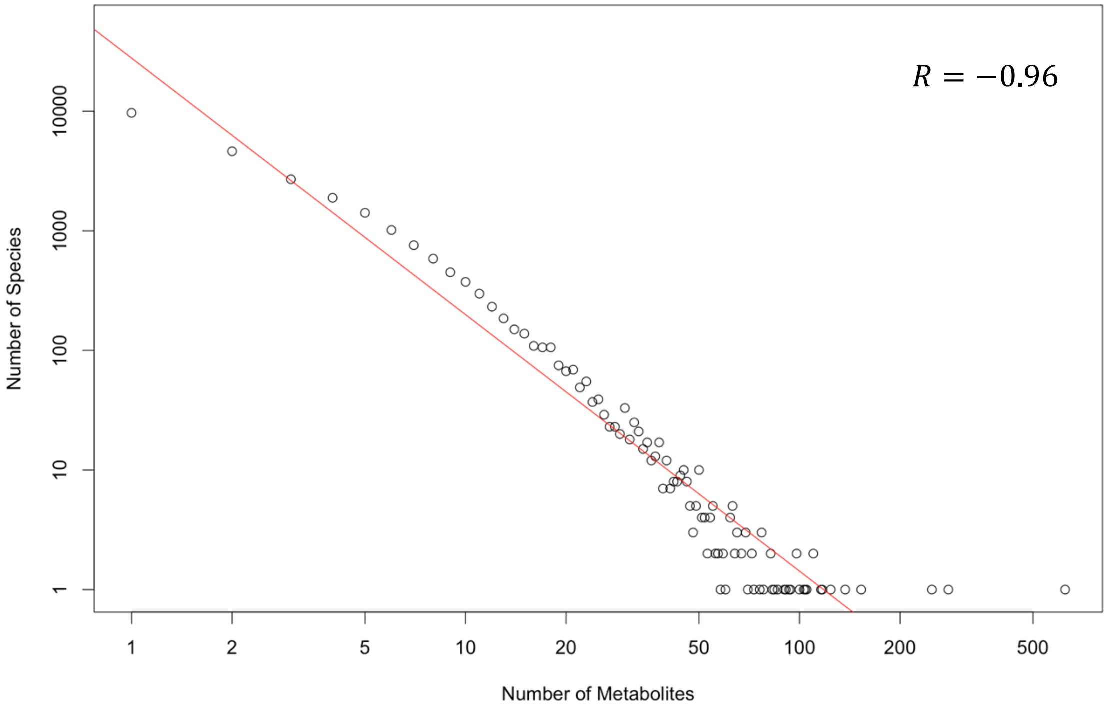

Supplementary Fig.1 The degree distribution of species in the species-metabolite bipartite graph. The x-axes represents the number of metabolites belonging to one species and the y-axes represents the frequency of such species.

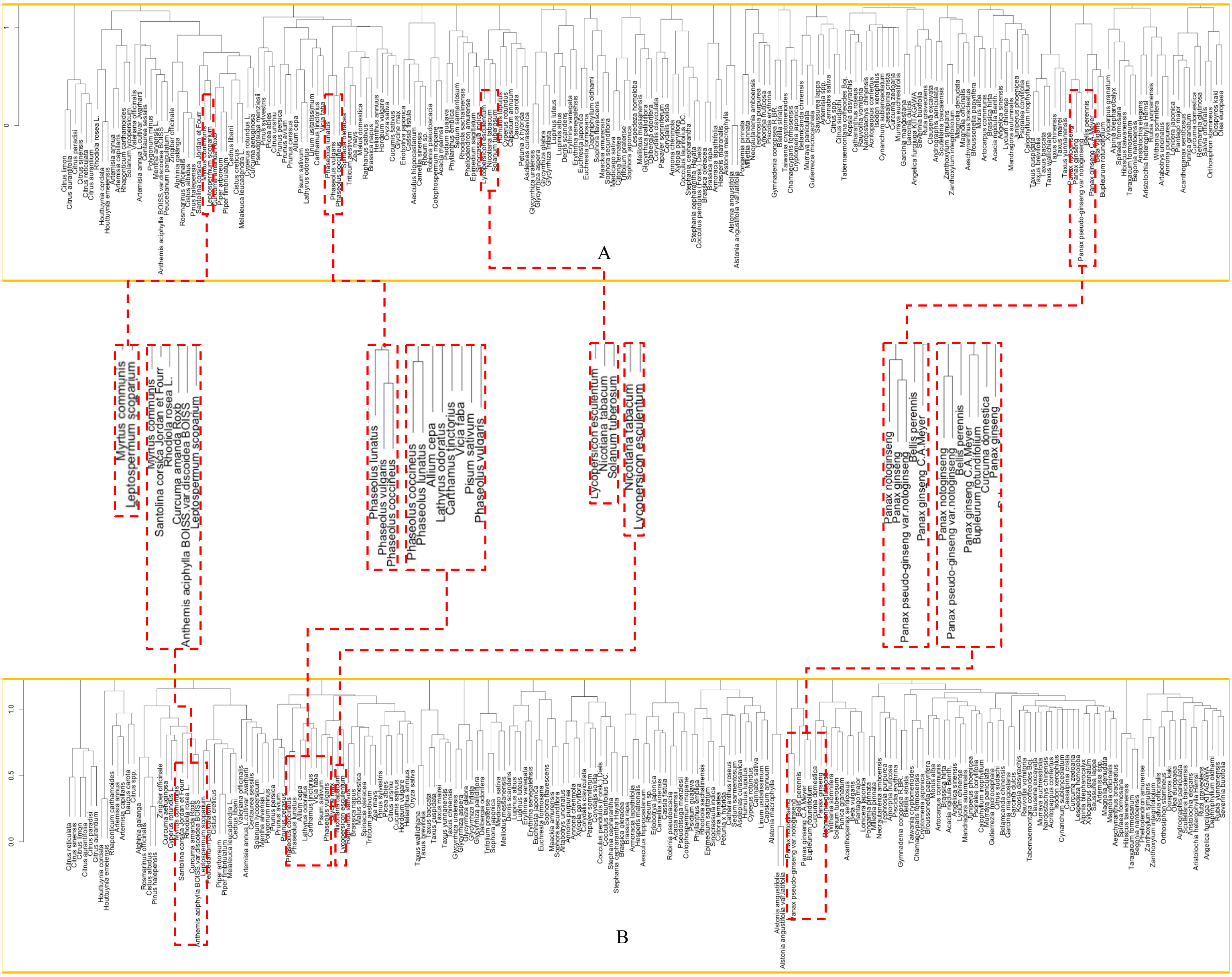

Supplementary Fig.2: (A) Hierarchical dendrogram plot of classification by Simpson similarity coefficients (B) Hierarchical dendrogram plot of classification by Jaccard similarity coefficients. *Myrtus communis* and *Leptospermum scoparium* belong to family *Myrtaceae*. *Phaseolus lunatus*, *Phaseolus vulgaris* and *Phaseolus coccineus* belong to genus *Phaseolus*. *Lycopersicon esculentum*, *Nicotiana tabacum* and *Solanum tuberosum* belong to family *Solanaceae*. *Panax notoginseng*, *Panax ginseng*, *Panax pseudo-ginseng var.notoginseng* and *Panax ginseng C.A.Meyer* belong to genus *Panax*. The Simpson coefficients based classification performs somewhat better.

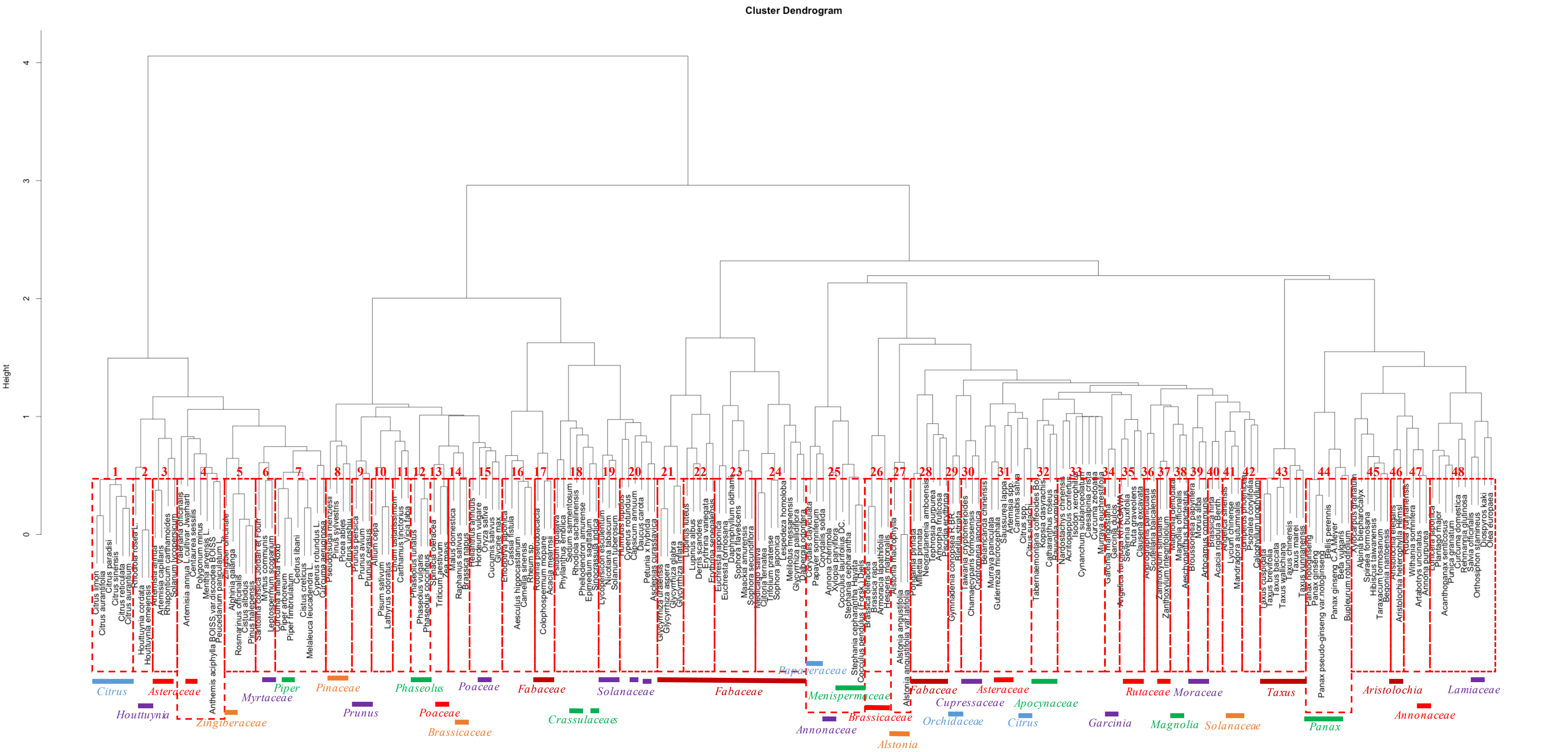

Supplementary Fig. 3: The *family* or *genus* level phylogeny patterns in proposed classification of 216 plants. Group IDs are annotated beside the corresponding plant groups. Adjacently placed plants of the same *genus* or *family* are indicated by thick bars of specific colors.

Supplementary Table 1. The predicted unrecorded plant-metabolite relations, involving 524 metabolites and 117 plants.

metabolite	plant
Gibberellin A4	*Citrus limon; Phaseolus lunatus; Nicotiana tabacum; Lupinus luteus*
Methyl salicylate	*Citrus limon; Citrus aurantifolia; Citrus paradisi; Citrus aurantium*
Cyclohexane	*Citrus limon; Citrus aurantium; Artemisia annua; Artemisia capillaris; Centaurea sessilis; Zingiber officinale*
o-Isopropenyl toluene	*Citrus limon; Citrus aurantifolia; Citrus paradisi; Citrus sinensis; Citrus aurantium*
Jasmonic acid	*Citrus limon*
10'-Apoviolaxanthal	*Citrus limon*
alpha-trans-Bergamotene	*Citrus limon*
Citral	*Citrus aurantifolia; Citrus paradisi; Citrus reticulata*
Benzeneacetaldehyde	*Citrus aurantifolia; Citrus paradisi; Citrus aurantium*
Methyl epijasmonate	*Citrus aurantifolia; Citrus sinensis*
Salvigenin	*Citrus aurantifolia; Citrus sinensis; Citrus aurantium*
Rhoifolin	*Citrus paradisi*
Isopropanol	*Citrus paradisi; Citrus reticulata*
Isoscutellarein 7,8-dimethyl ether	*Citrus sinensis*
Isoscutellarein 7,8,4'-trimethyl ether	*Citrus sinensis*
Gibberellin A53	*Citrus sinensis; Lathyrus odoratus; Phaseolus lunatus; Phaseolus coccineus; Hordeum vulgare; Nicotiana tabacum*
Violaxanthin	*Citrus sinensis*
Gibberellin A81	*Citrus reticulata*
Gibberellin A9	*Citrus reticulata*
6-Demethoxytangeritin	*Citrus reticulata*
Tetramethylscutellarein	*Citrus reticulata*
Apigenin 7-rutinoside	*Citrus aurantium*
Caryophyllene oxide	*Houttuynia emeiensis*
Capillaridin E	*Artemisia annua; Rhaponticum carthamoides*
Limonene	*Artemisia annua; Artemisia capillaris; Artemisia annua L.cultivar Jwarharti*
Cyclosativene	*Artemisia annua*
2-Nonanone	*Artemisia annua*
Kaempferol	*Artemisia capillaris; Sedum sarmentosum; Medicago sativa; Cryptomeria japonica*
Salicylic acid	*Artemisia capillaris*
Apigenin	*Artemisia capillaris*
Octanal	*Artemisia capillaris*
Rhamnocitrin	*Rhaponticum carthamoides*
Genkwanin	*Rhaponticum carthamoides*
p-Cymene	*Rhaponticum carthamoides*
Hexahydrofarnesyl acetone	*Artemisia annua L.cultivar Jwarharti*

Caprylic acid	*Centaurea sessilis*
Isophytol	*Centaurea sessilis; Anthemis aciphylla*
Phytol	*Centaurea sessilis; Anthemis aciphylla*
Bicyclogermacren	*Centaurea sessilis*
(2E)-Octenal	*Centaurea sessilis*
R-(+)-trans-Verbenol	*Centaurea sessilis*
2-Pentylfuran	*Anthemis aciphylla*
Isovaleraldehyde	*Alphinia galanga*
alpha-Thujene	*Leptospermum scoparium*
Neryl acetate	*Leptospermum scoparium*
6,9-Guaiadiene	*Piper arboreum*
Geraniol	*Piper arboreum*
Germacrene D	*Piper fimbriulatum*
Ampelopsin 7-glucoside	*Pseudotsuga menziesii*
Indole-3-carboxylic acid	*Pseudotsuga menziesii; Picea abies; Lycopersicon esculentum*
Gibberellin A9	*Pinus sylvestris; Lathyrus odoratus; Brassica napus; Hordeum vulgare*
Gibberellin A1	*Pinus sylvestris; Lycopersicon esculentum; Lupinus luteus*
Gibberellin A7	*Pinus sylvestris*
Indole-3-acetic acid	*Pinus sylvestris; Brassica rapa*
Methyl indole-3-acetate	*Pinus sylvestris*
Syringetin 3-(6''-acetylglucoside)	*Pinus sylvestris*
Laricitrin 3-(6''-acetylglucoside)	*Pinus sylvestris*
Taxifolin 3'-glucoside	*Picea abies*
Isorhamnetin 3-(6''-acetylgalactoside)	*Picea abies*
Gibberellin A20	*Prunus persica; Lycopersicon esculentum*
Afzelechin	*Prunus avium*
Gibberellin A44	*Prunus avium; Hordeum vulgare*
Gibberellin A30	*Prunus avium*
Chrysin	*Prunus avium*
Peonidin 3-rhamnoside	*Pisum sativum*
Gibberellin A97	*Lathyrus odoratus; Zea mays; Oryza sativa*
Gibberellin A51	*Lathyrus odoratus; Hordeum vulgare*
Gibberellin A12	*Lathyrus odoratus; Triticum aestivum; Brassica napus*
Gibberellin A24	*Lathyrus odoratus; Brassica napus; Hordeum vulgare*
Gibberellin A44	*Lathyrus odoratus*
Isosojagol	*Phaseolus lunatus*
Isoferreirin	*Phaseolus lunatus; Phaseolus vulgaris*
7,4'-Dihydroxy-5,2'-dimethoxyisoflavanone	*Phaseolus lunatus; Phaseolus vulgaris*
5-Deoxykievitol	*Phaseolus vulgaris*

(-)-Glycinol	*Phaseolus vulgaris*
Psoralidin	*Phaseolus coccineus*
Gibberellin A19	*Phaseolus coccineus; Lycopersicon esculentum*
Gibberellin A37	*Phaseolus coccineus*
Gibberellin A14	*Triticum aestivum*
Gibberellin A25	*Zea mays; Brassica napus*
Neoglucobrassicin	*Raphanus sativus; Armoracia lapathifolia*
Gibberellin A53	*Raphanus sativus*
Gibberellin A40	*Brassica napus*
Gibberellin A34	*Hordeum vulgare*
Gibberellin A8	*Oryza sativa*
Fisetin 3-methyl ether	*Robinia pseudoacacia*
Garbanzol	*Robinia pseudoacacia*
Butin	*Robinia pseudoacacia*
Gallocatechin	*Colophospermum mopane*
(+)-Gallocatechin	*Colophospermum mopane*
Dihydrorobinetin	*Acacia mearnsii*
Dihydroquercetin	*Acacia mearnsii*
Liquiritigenin	*Acacia mearnsii*
(-)-Epicatechin	*Acacia mearnsii*
ent-Epifisetinidol	*Acacia mearnsii*
Kaempferide	*Rhodiola sachalinensis; Sinocrassula indica*
Sinocrassoside C1	*Rhodiola sachalinensis*
Rhodionin	*Sinocrassula indica*
Rhodiosin	*Sinocrassula indica*
Nicotinic acid	*Lycopersicon esculentum*
9-Ribosyl-cis-zeatin	*Lycopersicon esculentum*
Indole-3-acetonitrile	*Nicotiana tabacum; Solanum tuberosum*
Trigonelline	*Nicotiana tabacum*
Phytuberin	*Nicotiana tabacum*
(-)-Phytuberin	*Nicotiana tabacum*
1-Caffeoyl-beta-D-glucose	*Nicotiana tabacum*
Desacetylphytuberin	*Solanum tuberosum*
9-Ribosyl-trans-zeatin	*Solanum tuberosum*
Prunetin	*Glycyrrhiza uralensis; Medicago sativa; Clitoria ternatea; Melilotus messanensis; Glycyrrhiza pallidiflora; Dalbergia odorifera*
Licoisoflavone A	*Glycyrrhiza uralensis; Glycyrrhiza inflata*
Glabridin	*Glycyrrhiza uralensis*
Topazolin	*Glycyrrhiza uralensis*
Isoderrone	*Glycyrrhiza uralensis; Glycyrrhiza aspera*
Licoflavone B	*Glycyrrhiza uralensis*

3'-Dimethylallylkievitone	*Glycyrrhiza uralensis*
Wighteone	*Glycyrrhiza uralensis; Glycyrrhiza aspera; Lupinus luteus; Derris scandens*
Hydroxywighteone	*Glycyrrhiza uralensis; Glycyrrhiza aspera; Lupinus albus; Erythrina variegata*
Licopyranocoumarin	*Glycyrrhiza aspera; Glycyrrhiza inflata*
Kanzonol W	*Glycyrrhiza aspera*
Licuroside	*Glycyrrhiza aspera; Glycyrrhiza inflata*
Neoisoliquiritigenin	*Glycyrrhiza aspera; Glycyrrhiza inflata*
1-O-Methylglycyrol	*Glycyrrhiza aspera; Glycyrrhiza glabra*
Kanzonol P	*Glycyrrhiza aspera; Glycyrrhiza glabra*
Neoliquiritin	*Glycyrrhiza aspera; Glycyrrhiza glabra; Glycyrrhiza inflata*
Licoricone	*Glycyrrhiza aspera; Glycyrrhiza glabra; Glycyrrhiza inflata*
Licoricesaponin A3	*Glycyrrhiza aspera; Glycyrrhiza glabra*
Licoricesaponin G2	*Glycyrrhiza aspera; Glycyrrhiza glabra*
Licoricesaponin C2	*Glycyrrhiza aspera; Glycyrrhiza glabra; Glycyrrhiza inflata*
Licoricesaponin E2	*Glycyrrhiza aspera; Glycyrrhiza glabra; Glycyrrhiza inflata*
Licoflavonol	*Glycyrrhiza aspera*
Gancaonin Q	*Glycyrrhiza aspera; Glycyrrhiza inflata*
Glisoflavanone	*Glycyrrhiza aspera*
Kanzonol I	*Glycyrrhiza aspera; Glycyrrhiza glabra*
Glycyrrhizol A	*Glycyrrhiza aspera*
Licoriphenone	*Glycyrrhiza aspera*
Licofuranocoumarin	*Glycyrrhiza glabra; Glycyrrhiza inflata*
Glyasperin L	*Glycyrrhiza glabra; Glycyrrhiza inflata*
Licocoumarone	*Glycyrrhiza glabra; Glycyrrhiza inflata*
Glyinflanin E	*Glycyrrhiza glabra*
Glyinflanin F	*Glycyrrhiza glabra*
Gancaonin A	*Glycyrrhiza glabra*
Semilicoisoflavone B	*Glycyrrhiza inflata*
Licoagrochalcone D	*Glycyrrhiza inflata*
Pratensol	*Lupinus luteus; Derris scandens; Clitoria ternatea; Melilotus messanensis; Glycyrrhiza pallidiflora; Dalbergia odorifera*
Indicanin E	*Lupinus luteus; Lupinus albus; Derris scandens*
Derrisisoflavone B	*Lupinus luteus; Lupinus albus*
Lupinisol A	*Lupinus luteus*
Lupalbigenin	*Lupinus luteus*
Derrisisoflavone F	*Lupinus luteus; Lupinus albus*
Lupinalbin B	*Lupinus luteus*
Lupinalbin G	*Lupinus luteus*
Laburnetin	*Lupinus luteus; Lupinus albus*
3'-Methylorobol	*Lupinus luteus; Lupinus albus*
Genistein	*Lupinus albus; Melilotus messanensis; Glycyrrhiza pallidiflora; Dalbergia odorifera*

Isoprunetin	*Lupinus albus; Derris scandens*
Scanderone	*Lupinus albus*
Chandalone	*Lupinus albus*
Ulexone A	*Lupinus albus*
Isolupalbigenin	*Lupinus albus; Derris scandens*
Gibberellin A18	*Lupinus albus*
Gibberellin A23	*Lupinus albus*
Lupinol C	*Lupinus albus*
Scandenal	*Lupinus albus; Erythrina variegata*
Lupisoflavone	*Derris scandens*
Alpinumisoflavone	*Derris scandens*
Derrone	*Derris scandens; Erythrina variegata*
Angustone B	*Derris scandens*
Angustone C	*Derris scandens*
Lupinisolone A	*Derris scandens*
Lupinisoflavone H	*Derris scandens*
Lupinifolin	*Derris scandens*
Barpisoflavone C	*Erythrina variegata*
Erysenegalensein K	*Erythrina variegata*
8-Prenylluteone	*Erythrina variegata*
Robustic acid	*Erythrina variegata; Erythrina senegalensis*
Eturunagarone	*Erythrina variegata; Erythrina senegalensis*
Bidwillon B	*Erythrina senegalensis*
Lonchocarpic acid	*Erythrina senegalensis*
Lonchocarpenin	*Erythrina senegalensis*
Scandenin	*Erythrina senegalensis*
Euchretin E	*Euchresta japonica*
Euchretin D	*Euchresta japonica*
Euchretin M	*Euchresta japonica*
Cytisine	*Euchresta japonica; Sophora flavescens; Maackia amurensis*
Formononetin	*Euchresta formosana*
Secundiflorol I	*Euchresta formosana; Sophora flavescens; Maackia amurensis*
Kushenin	*Euchresta formosana; Maackia amurensis; Sophora secundiflora; Sophora japonica; Glycyrrhiza pallidiflora*
Euchrenone a4	*Euchresta formosana*
Amorilin	*Euchresta formosana*
(-)-N-Methylcytisine	*Euchresta formosana*
Daidzein	*Sophora flavescens; Maackia amurensis; Melilotus messanensis; Glycyrrhiza pallidiflora; Dalbergia odorifera*
Thermopsine	*Sophora flavescens; Maackia amurensis*
(-)-6alpha-Hydroxylupanine	*Sophora flavescens; Sophora secundiflora*

(-)-6alpha-methoxylupanine	*Sophora flavescens; Sophora secundiflora*
Pratensein	*Maackia amurensis*
Mamanine	*Maackia amurensis; Sophora secundiflora*
5,6-Dehydrolupanine	*Maackia amurensis*
Argentine	*Maackia amurensis*
Maackiain	*Sophora secundiflora*
(-)-12,12'-Methylenedicytisine	*Sophora secundiflora*
Soyasapogenol E	*Medicago sativa; Trifolium pratense*
Xenognosin B	*Medicago sativa; Clitoria ternatea; Trifolium pratense; Sophora japonica; Melilotus messanensis*
Vestitone	*Medicago sativa*
Anhydroglycinol	*Medicago sativa; Trifolium pratense; Melilotus messanensis*
Lespedezol A1	*Medicago sativa; Trifolium pratense; Melilotus messanensis*
6a-Hydroxymedicarpin	*Medicago sativa; Glycyrrhiza pallidiflora; Dalbergia odorifera*
Sophorophenolone	*Medicago sativa*
Delphinidine	*Medicago sativa; Trifolium pratense; Sophora japonica; Melilotus messanensis*
Quercetin	*Medicago sativa; Clitoria ternatea; Melilotus messanensis*
Lespedezol G1	*Medicago sativa*
Licoagroside C	*Medicago sativa*
Pratol	*Medicago sativa; Glycyrrhiza pallidiflora*
Licoagroside E	*Medicago sativa*
7,4'-Di-O-methyldaidzein	*Medicago sativa; Trifolium pratense; Melilotus messanensis; Glycyrrhiza pallidiflora; Dalbergia odorifera*
Licodione 2'-methyl ether	*Medicago sativa*
Tricin	*Clitoria ternatea; Trifolium pratense*
Myricetin	*Clitoria ternatea; Trifolium pratense; Sophora japonica*
Irisolidone	*Trifolium pratense; Glycyrrhiza pallidiflora*
Erythrinin C	*Trifolium pratense; Sophora japonica*
Licoagroisoflavone	*Trifolium pratense; Sophora japonica*
Kushenin	*Trifolium pratense*
Tectoridin	*Trifolium pratense*
Sophojaponicin	*Trifolium pratense; Glycyrrhiza pallidiflora*
7,3'-Dimethylorobol	*Trifolium pratense*
Irisolidone 7-O-beta-D-glucoside	*Trifolium pratense*
(-)-Maackiain	*Trifolium pratense; Lespedeza homoloba*
Irilone	*Sophora japonica; Glycyrrhiza pallidiflora*
Isokaempferide	*Sophora japonica*
Trifoliol	*Sophora japonica*
Trifolirhizin	*Sophora japonica*
Irilone 4'-O-glucoside	*Sophora japonica*
(+)-6a-Hydroxymaackiain	*Sophora japonica; Lespedeza homoloba; Glycyrrhiza pallidiflora*

Coumestrol	*Lespedeza homoloba*
Bolusanthin III	*Lespedeza homoloba*
9-O-Methylcoumestrol	*Lespedeza homoloba*
5'-Methoxysativan	*Lespedeza homoloba*
Sativanone	*Melilotus messanensis*
2-Methoxymedicarpin	*Melilotus messanensis*
Coumestrin	*Glycyrrhiza pallidiflora*
Haginin D	*Glycyrrhiza pallidiflora*
Lespedezol F1	*Glycyrrhiza pallidiflora*
2'-Methoxyisoliquiritigenin	*Glycyrrhiza pallidiflora*
Echinatin	*Dalbergia odorifera*
Glypallichalcone	*Dalbergia odorifera*
Melilotocarpan B	*Dalbergia odorifera*
Cryptopine	*Corydalis claviculata; Corydalis solida*
Allocryptopine	*Corydalis claviculata; Papaver somniferum*
Rhoeadine	*Corydalis claviculata*
Norribasine	*Papaver somniferum*
Ribasine	*Papaver somniferum*
Sinoacutine	*Papaver somniferum*
alpha-Hydrastine	*Papaver somniferum*
Noscapine	*Corydalis solida*
Narcotoline	*Corydalis solida*
Salutaridine	*Corydalis solida*
Oxocularicine	*Corydalis solida*
Glaziovine	*Annona cherimola; Cocculus laurifolius; Artabotrys uncinatus*
Calycinine	*Annona cherimola*
Juzirine	*Annona cherimola*
Pronuciferine	*Xylopia parviflora; Stephania cepharantha Hayata*
Annocherine A	*Xylopia parviflora*
Annocherine B	*Xylopia parviflora*
Michelalbine	*Xylopia parviflora*
Micheline A	*Xylopia parviflora*
14-Episinomenine	*Cocculus laurifolius; Stephania cepharantha*
Cephamonine	*Stephania cepharantha*
Aknadilactam	*Stephania cepharantha*
Stephodeline	*Stephania cepharantha*
Tannagine	*Stephania cepharantha*
Aknadinine	*Stephania cepharantha*
Juziphine	*Stephania cepharantha*
N-Methylasimilobine	*Stephania cepharantha*
Coclaurine	*Stephania cepharantha Hayata*

Anonaine	*Stephania cepharantha Hayata*
Aknadicine	*Stephania cepharantha Hayata*
Cucoline	*Stephania cepharantha Hayata*
Oblongine	*Stephania cepharantha Hayata*
1-Methoxybrassitin	*Brassica oleracea*
Glucoviorylin	*Brassica oleracea; Brassica rapa; Hesperis matronalis*
Glucolepidiin	*Brassica oleracea; Brassica rapa; Hesperis matronalis*
Caulilexin C	*Brassica oleracea*
Brassicanal B	*Brassica oleracea*
Benzylglucosinolate	*Brassica oleracea; Brassica rapa; Hesperis matronalis*
Glucocheirolin	*Brassica oleracea; Brassica rapa; Hesperis matronalis*
Spirobrassinin	*Brassica oleracea*
3-Hydroxybutyl glucosinolate	*Brassica oleracea; Brassica rapa; Hesperis matronalis*
4-Methoxyglucobrassicin	*Brassica oleracea*
Sinigrin	*Brassica rapa; Hesperis matronalis*
Methyl anthranilate	*Brassica rapa*
Glucoberteroin	*Brassica rapa*
1-Methoxyspirobrassinin	*Brassica rapa*
Glucosinalbate	*Armoracia lapathifolia*
Glucoalyssin	*Armoracia lapathifolia*
Glucohesperin	*Armoracia lapathifolia*
Glucoiberin	*Hesperis matronalis*
Glucobrassicanapin	*Hesperis matronalis*
Glucolesquerellin	*Hesperis matronalis*
2-Methoxybenzyl glucosinolate	*Hesperis matronalis*
Alstolactone	*Alstonia macrophylla*
Isoalstonisine	*Alstonia macrophylla*
Normacusine B	*Alstonia macrophylla*
N(4)-Demethylalstonerinal	*Alstonia macrophylla; Alstonia angustifolia*
Cathafoline N(4)-oxide	*Alstonia macrophylla*
Alstophyllal	*Alstonia angustifolia; Alstonia angustifolia var.latifolia*
Macrocarpine A	*Alstonia angustifolia; Alstonia angustifolia var.latifolia*
Macrocarpine B	*Alstonia angustifolia; Alstonia angustifolia var.latifolia*
Alstohentine	*Alstonia angustifolia; Alstonia angustifolia var.latifolia*
Alstonisine	*Alstonia angustifolia*
Alstomaline	*Alstonia angustifolia; Alstonia angustifolia var.latifolia*
N1-Demethylalstophylline	*Alstonia angustifolia; Alstonia angustifolia var.latifolia*
(-)-Vincamajine	*Alstonia angustifolia var.latifolia*
Isopongaflavone	*Pongamia pinnata; Tephrosia purpurea*
Pongaglabol methyl ether	*Pongamia pinnata; Tephrosia purpurea*
Kanjone	*Pongamia pinnata; Tephrosia purpurea*

Lanceolatin B	*Pongamia pinnata*
Glabranin	*Pongamia pinnata*
Purpurenone	*Millettia pinnata*
O-Methylpongamol	*Millettia pinnata*
Purpuritenin B	*Millettia pinnata*
Pinnatin	*Millettia pinnata*
Piscisoflavone D	*Millettia pinnata*
Praecansone B	*Millettia pinnata*
Ovalichromene B	*Millettia pinnata*
Piscisoflavone A	*Neorautanenia amboensis*
Millettone	*Neorautanenia amboensis*
Pongachalcone I	*Tephrosia purpurea*
Pongapinnol D	*Tephrosia purpurea*
Karanjachromene	*Tephrosia purpurea*
Dihydroamorphigenin	*Tephrosia purpurea; Piscidia erythrina*
Dalpanol	*Tephrosia purpurea; Piscidia erythrina*
Amorphigenol	*Tephrosia purpurea; Piscidia erythrina*
Amorphigenin	*Tephrosia purpurea; Piscidia erythrina*
Jamaicin	*Tephrosia purpurea; Amorpha fruticosa*
Ichthynone	*Tephrosia purpurea; Amorpha fruticosa*
Dehydromillettone	*Tephrosia purpurea; Amorpha fruticosa*
3-O-Demethylamorphigenin	*Tephrosia purpurea; Piscidia erythrina*
Rotenone	*Amorpha fruticosa*
Candidone	*Piscidia erythrina*
Dehydrodeguelin	*Piscidia erythrina*
Ambofuranol	*Piscidia erythrina*
Neorautenanol	*Piscidia erythrina*
Isopongachromene	*Piscidia erythrina*
Blestrianol A	*Gymnadenia conopsea*
Blestrin A	*Gymnadenia conopsea*
Blestrin B	*Gymnadenia conopsea*
Blestrin C	*Gymnadenia conopsea*
Blestrin D	*Gymnadenia conopsea*
Isoarundinin-II	*Gymnadenia conopsea*
Bulbocodin C	*Bletilla striata*
Bulbocodin D	*Bletilla striata*
Gymconopin C	*Bletilla striata*
Bulbocol	*Bletilla striata*
Gymconopin D	*Bletilla striata*
12-Methylferruginol	*Taiwania cryptomerioides*
Pisiferal	*Taiwania cryptomerioides*

(-)-Nortrachelogenin	*Taiwania cryptomerioides; Cryptomeria japonica*
Matairesinol	*Chamaecyparis formosensis*
Cryptomeridiol	*Chamaecyparis formosensis*
Cubeb camphor	*Chamaecyparis formosensis*
Epicubebol	*Chamaecyparis formosensis*
Diphyllin	*Chamaecyparis formosensis*
Sandaracopimarinal	*Chamaecyparis formosensis*
Sugiol	*Chamaecyparis formosensis*
3,7,4'-Tri-O-methylkaempferol	*Chamaecyparis formosensis*
19-Hydroxyferruginol	*Chamaecyparis formosensis*
Pisiferanol	*Cryptomeria japonica*
Pisiferol	*Cryptomeria japonica*
Chaenocephalol	*Cryptomeria japonica*
(-)-Pluviatolide	*Cryptomeria japonica*
12-O-Methylpisiferanol	*Cryptomeria japonica*
alpha-Cadinol	*Cryptomeria japonica*
Sugiol methyl ether	*Cryptomeria japonica*
(+)-beta-Cyclocostunolide	*Artemisia spp.*
Arbusculin C	*Saussurea lappa*
Nortetraphyllicine	*Tabernaemontana coffeoides*
Vincamine	*Kopsia dasyrachis*
Ajmalicine	*Kopsia dasyrachis; Rauvolfia vomitoria*
Sarpagine	*Catharanthus roseus*
Alstonine	*Catharanthus roseus*
(+)-Isoeburnamine	*Catharanthus roseus*
(+)-Eburnamonine	*Catharanthus roseus*
14,15-Dihydroxyvincadifformine	*Catharanthus roseus*
Serpentine	*Rauvolfia vomitoria*
10-O-Methylsarpagine	*Rauvolfia vomitoria*
Dulcisxanthone B	*Garcinia mangostana*
Cowaxanthone D	*Garcinia mangostana*
Isonormangostin	*Garcinia mangostana*
Normangostin	*Garcinia dulcis*
Garcinone C	*Garcinia dulcis*
Mangostenone D	*Garcinia dulcis*
Mangostenone E	*Garcinia dulcis*
Tovophyllin B	*Garcinia dulcis*
Arborinine	*Severinia buxifolia*
Xanthoxyletin	*Ruta graveolens*
Clausamine A	*Ruta graveolens*
1,2,3-Trihydroxyacridone	*Ruta graveolens*

Bergapten	*Clausena excavata*
Psoralen	*Clausena excavata*
Rutacridone	*Clausena excavata*
Isoscopoletin	*Zanthoxylum simulans*
N-Methylflindersine	*Zanthoxylum simulans*
(-)-5-Methoxybalanophonin	*Zanthoxylum simulans*
beta-Sitosterone	*Zanthoxylum integrifoliolum*
Sepesteonol	*Zanthoxylum integrifoliolum*
Zanthobungeanine	*Zanthoxylum integrifoliolum*
Flindersine	*Zanthoxylum integrifoliolum*
Scopoletin	*Broussonetia papyrifera*
Sanggenon L	*Broussonetia papyrifera*
Gemichalcone B	*Broussonetia papyrifera*
Gemichalcone C	*Broussonetia papyrifera*
Artocommunol CA	*Morus alba*
Artocommunol CE	*Morus alba*
Marmesin	*Morus alba*
Dihydrocycloartomunin	*Morus alba*
Dihydroisocycloartomunin	*Morus alba*
Cycloartomunin	*Morus alba*
Artochamin D	*Morus alba*
Broussoflavonol A	*Morus alba*
(-)-Cycloartocarpin	*Morus alba*
Morusin	*Artocarpus communis*
Sanggenon M	*Artocarpus communis*
Cyclomulberrin	*Artocarpus communis*
Sanggenon A	*Artocarpus communis*
Mulberrin	*Artocarpus communis*
Demethoxyisogemichalcone C	*Artocarpus communis*
Isogemichalcone C	*Artocarpus communis*
Calystegin B2	*Lycium chinense*
Atropine	*Lycium chinense*
Calystegine B4	*Mandragora autumnalis*
Baccatin V	*Taxus cuspidata; Taxus brevifolia; Taxus wallichiana; Taxus chinensis; Taxus mairei; Taxus yunnanensis*
Taxol D	*Taxus cuspidata; Taxus chinensis; Taxus yunnanensis*
Dantaxusin D	*Taxus cuspidata; Taxus baccata; Taxus chinensis; Taxus mairei*
9-Deacetyltaxinine	*Taxus cuspidata; Taxus chinensis; Taxus mairei; Taxus yunnanensis*
Taxchin B	*Taxus cuspidata; Taxus baccata; Taxus mairei; Taxus yunnanensis*
Taxinine B	*Taxus cuspidata; Taxus chinensis*
Taxine I	*Taxus cuspidata; Taxus chinensis*

Taxumairol A	*Taxus cuspidata; Taxus baccata; Taxus chinensis*
Taxchinin H	*Taxus cuspidata; Taxus brevifolia*
Taxol B	*Taxus cuspidata*
Dantaxusin A	*Taxus cuspidata; Taxus baccata; Taxus chinensis; Taxus mairei*
Taxayuntin E	*Taxus cuspidata; Taxus chinensis*
Taxayuntin F	*Taxus cuspidata*
Taxchinin A	*Taxus cuspidata*
Taxchinin D	*Taxus cuspidata; Taxus baccata; Taxus wallichiana*
Taxchinin C	*Taxus cuspidata; Taxus brevifolia; Taxus yunnanensis*
Taxchinin K	*Taxus cuspidata; Taxus brevifolia; Taxus yunnanensis*
Taxayuntin H	*Taxus cuspidata; Taxus brevifolia; Taxus baccata; Taxus wallichiana; Taxus mairei*
Baccatin IV	*Taxus cuspidata; Taxus yunnanensis*
Taxamairin B	*Taxus cuspidata*
Dantaxusin C	*Taxus cuspidata; Taxus baccata; Taxus mairei*
Taxinine A	*Taxus cuspidata*
7-Deacetylcanadensene	*Taxus cuspidata; Taxus baccata*
Chinentaxunine	*Taxus cuspidata*
N-Methyltaxol C	*Taxus cuspidata; Taxus chinensis; Taxus yunnanensis*
14beta-benzoyloxybaccatin IV	*Taxus cuspidata; Taxus baccata; Taxus mairei*
5-Decinnamoyltaxuspine D	*Taxus cuspidata; Taxus yunnanensis*
5-Cinnamoyltaxicin I triacetate	*Taxus cuspidata; Taxus chinensis; Taxus mairei; Taxus yunnanensis*
5-epi-Canadensene	*Taxus cuspidata; Taxus chinensis; Taxus mairei*
7,2'-Bisdeacetoxyaustropicatine	*Taxus cuspidata*
Taxuspinanane I	*Taxus brevifolia; Taxus baccata; Taxus wallichiana; Taxus chinensis; Taxus mairei; Taxus yunnanensis*
Ormosin VI	*Taxus brevifolia; Taxus mairei*
Taxchinin B	*Taxus brevifolia; Taxus wallichiana*
Taxuspine V	*Taxus brevifolia; Taxus mairei*
14beta-Hydroxytaxusin	*Taxus brevifolia; Taxus baccata; Taxus wallichiana*
Epitaxol	*Taxus baccata; Taxus wallichiana; Taxus chinensis; Taxus mairei; Taxus yunnanensis*
Taxachitriene A	*Taxus baccata*
Taxachitriene B	*Taxus baccata*
Taxuspine S	*Taxus baccata; Taxus chinensis; Taxus mairei*
Taxezopidine L	*Taxus baccata; Taxus mairei*
Taxol C	*Taxus baccata*
Taxuspinanane A	*Taxus baccata; Taxus chinensis; Taxus yunnanensis*
Taxinine E	*Taxus baccata; Taxus chinensis*
Taxuspine Z	*Taxus baccata; Taxus wallichiana*
Taxezopidine E	*Taxus baccata*
Taxuspine X	*Taxus baccata*
Taxezopidine F	*Taxus baccata; Taxus yunnanensis*

Taxuyunnanine C	*Taxus baccata*
Taxuspinanane B	*Taxus baccata; Taxus wallichiana; Taxus chinensis*
Ponasterone A	*Taxus baccata; Taxus chinensis*
Decinnamoyltaxinine E	*Taxus baccata*
Taxezopidine H	*Taxus baccata; Taxus mairei*
Taxuspine D	*Taxus baccata; Taxus mairei*
Taxin B	*Taxus baccata*
Taxuspine U	*Taxus baccata; Taxus mairei*
5-Deacetyltaxachitriene B	*Taxus baccata*
2-Deacetyltaxachitriene A	*Taxus baccata*
Taxuspine A	*Taxus wallichiana; Taxus chinensis*
Taxuspine P	*Taxus wallichiana; Taxus yunnanensis*
Isolariciresinol	*Taxus wallichiana; Taxus yunnanensis*
Taxuspine T	*Taxus chinensis; Taxus mairei*
Taxine II	*Taxus chinensis*
Taxuspine B	*Taxus chinensis; Taxus mairei*
Taxuspine E	*Taxus chinensis; Taxus yunnanensis*
Taxayuntin J	*Taxus chinensis; Taxus mairei*
Taxumairol F	*Taxus chinensis; Taxus yunnanensis*
Deaminoacyltaxine A	*Taxus chinensis; Taxus mairei; Taxus yunnanensis*
Isotaxine B	*Taxus chinensis; Taxus yunnanensis*
Taxinine H	*Taxus chinensis; Taxus mairei*
Dantaxusin B	*Taxus chinensis; Taxus mairei*
Taxayuntin A	*Taxus chinensis*
2-Deacetyldecinnamoyltaxinine E	*Taxus chinensis; Taxus mairei*
Taxuyunnanine N	*Taxus mairei*
10-Deacetyltaxol A	*Taxus mairei*
13-Deacetoxybaccatin I	*Taxus mairei; Taxus yunnanensis*
Taxumairol V	*Taxus yunnanensis*
Taxchinin I	*Taxus yunnanensis*
Methyl pyroglutamate	*Panax notoginseng*
Ginsenoside Rh7	*Panax notoginseng; Panax pseudo-ginseng var.notoginseng*
Ginsenoyne E	*Panax ginseng; Panax pseudo-ginseng var.notoginseng*
Notoginsenoside	*Panax ginseng*
Panaxydol	*Panax pseudo-ginseng var.notoginseng*
Ginsenoside Re	*Panax pseudo-ginseng var.notoginseng*
Aristolactam IIIa	*Aristolochia elegans*
Aristolochate I	*Aristolochia elegans*
Aristolactam I	*Aristolochia elegans*
Methyl aristolochate	*Aristolochia elegans*
Aristolochate C	*Aristolochia elegans*

Aristolochic acid I	*Aristolochia heterophylla*
4-Hydroxybenzoic acid	*Aristolochia heterophylla*
O-Methylflavinantine	*Artabotrys uncinatus*
Pallidine	*Artabotrys uncinatus*
Norpallidine	*Artabotrys uncinatus*
Lysicamine	*Artabotrys uncinatus*
Flavinantine	*Annona purpurea*
